# Mechanisms of fusidic acid resistance

**DOI:** 10.1042/BST20253064

**Published:** 2025-08-18

**Authors:** Adrián González-López, Maria Selmer

**Affiliations:** 1 Department of Cell and Molecular Biology, Uppsala University, BMC, P.O. Box 596, SE-75124 Uppsala, Sweden; 2 Uppsala Antibiotic Center, Uppsala University, Sweden

**Keywords:** fusidic acid, ribosome, elongation factor G, FusB, antibiotic resistance

## Abstract

Fusidic acid (FA) is an antibiotic used to treat staphylococcal infections, particularly *Staphylococcus aureus*. It acts by inhibiting protein synthesis through locking elongation factor G (EF-G) to the ribosome. In *S. aureus*, there are three mechanisms of resistance. Mutations in the antibiotic target, EF-G (*fusA*), are common. These mutations affect the FA binding or the stability of the FA-locked state of EF-G but, due to effects on the normal function of EF-G, impose a fitness cost for the pathogen. The most common mechanism, FusB-type, involves expression of a resistance protein, FusB or FusC (FusD or FusF in other staphylococci), that provides target protection. The resistance protein binds to EF-G in its FA-locked state and mediates its release from the ribosome. An uncommon resistance mechanism (FusE) involves mutations in a ribosomal protein, uL6. In other bacteria, outside of its current clinical use, resistance to FA involves efflux pumps, limited membrane permeability, or enzymes that chemically alter FA. On a global level, the prevalence of FA resistance is relatively low, indicating that the antibiotic remains effective.

## Introduction

Antibiotic resistance is a global problem, having caused an estimated 4.71 million associated deaths in 2021 and predicted to increase to more than 8 million per year by 2050 [1]. It is important to understand the underlying molecular mechanisms of resistance as a foundation for future discovery of countermeasures.

**Figure 1:**
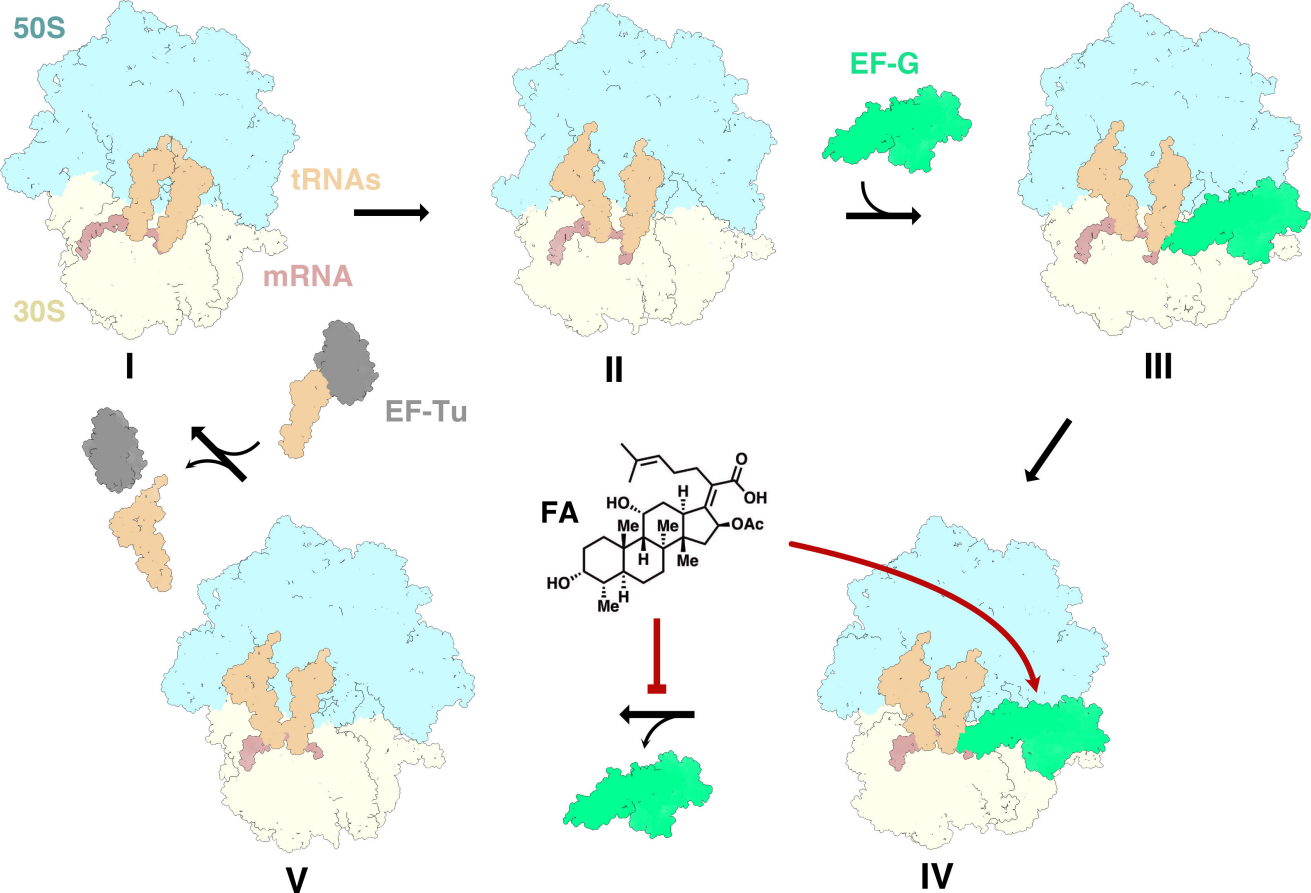
Elongation cycle of bacterial protein synthesis and inhibition by FA**.** (**I**) Aminoacyl-tRNA is delivered by EF-Tu and the peptidyl-transfer reaction occurs. (**II**) The ribosome rotates and the tRNAs move with respect to the 50S subunit. (**III**) EF-G binds to the rotated ribosome. (**IV**) EF-G hydrolyzes GTP and translocates the mRNA and tRNA with respect to the 30S subunit. FA can bind. (**V**) EF-G dissociates from the ribosome. This step is inhibited by FA. The ribosome is colored by subunit (30S, yellow and 50S, blue), mRNA in brown, tRNAs in beige, EF-G in green and EF-Tu in gray.

Fusidic acid (FA) is a steroid antibiotic (Figure 1) produced by the fungus *Acremonium fusidioides* (previously called *Fusidium coccineum*) [2], using a biosynthetic gene cluster consisting of eight genes [3]. Clinical use of FA was introduced in 1962 [4] as a narrow-spectrum bacteriostatic antibiotic targeting protein synthesis. It is the only clinically used antibiotic of the fusidane class [3], primarily effective against Gram-positive bacteria, and used toward staphylococci, such as *Staphylococcus aureus* and *Staphylococcus epidermidis* with minimum inhibitory concentrations (MICs) of 0.03–0.25 µg/ml [5]. According to the European Committee on Antimicrobial Susceptibility Testing in 2025, *Staphylococcus* spp*.* are considered resistant to FA when the MIC is above 1 µg/ml (available at https://www.eucast.org/).

*S. aureus* is a top priority pathogen according to the World Health Organization [6] due to its high rate of methicillin resistance, with median reported rates of 35% according to the 2022 Global Antimicrobial Resistance and Use Surveillance System [7]. In addition, methicillin-resistant *S. aureus* (MRSA) is reported to be the deadliest resistant pathogen globally in 2021 [1]. Alternative antibiotics against MRSA are thus of high relevance.

The focus of this mini-review is to describe the most recent progress on understanding the mechanisms of resistance to FA and their prevalence.

### Mechanism of action of FA 

Protein synthesis is the cellular process through which mRNA is translated into protein. It involves several steps (initiation, elongation, termination, and ribosome recycling), which can all be inhibited by antibiotics that bind to the ribosome or to the protein factors that assist the respective steps [8]. FA targets the GTPase elongation factor G (EF-G). During elongation, EF-G catalyzes translocation, the movement of tRNAs and mRNA by one three-base codon to allow binding of a new aminoacyl tRNA to the next codon on the ribosome [9] (Figure 1). In addition, EF-G also catalyzes ribosome recycling, the step where the ribosomal subunits are split for use in a new translation cycle after termination and release of the nascent peptide [10]. Binding of FA to EF-G prevents EF-G release from the ribosome, blocking the ribosomal A-site, thereby preventing delivery of the next aminoacyl tRNA (Figure 1) and the continued translation cycle. *In vitro,* FA has been shown to inhibit EF-G both in translocation [11,12] and ribosome recycling [13,14]. While the main bacteriostatic effect has been linked to inhibition of translocation, the inhibition of ribosome recycling was suggested to have a significant impact on the translation of short open reading frames [14].

**Figure 2:**
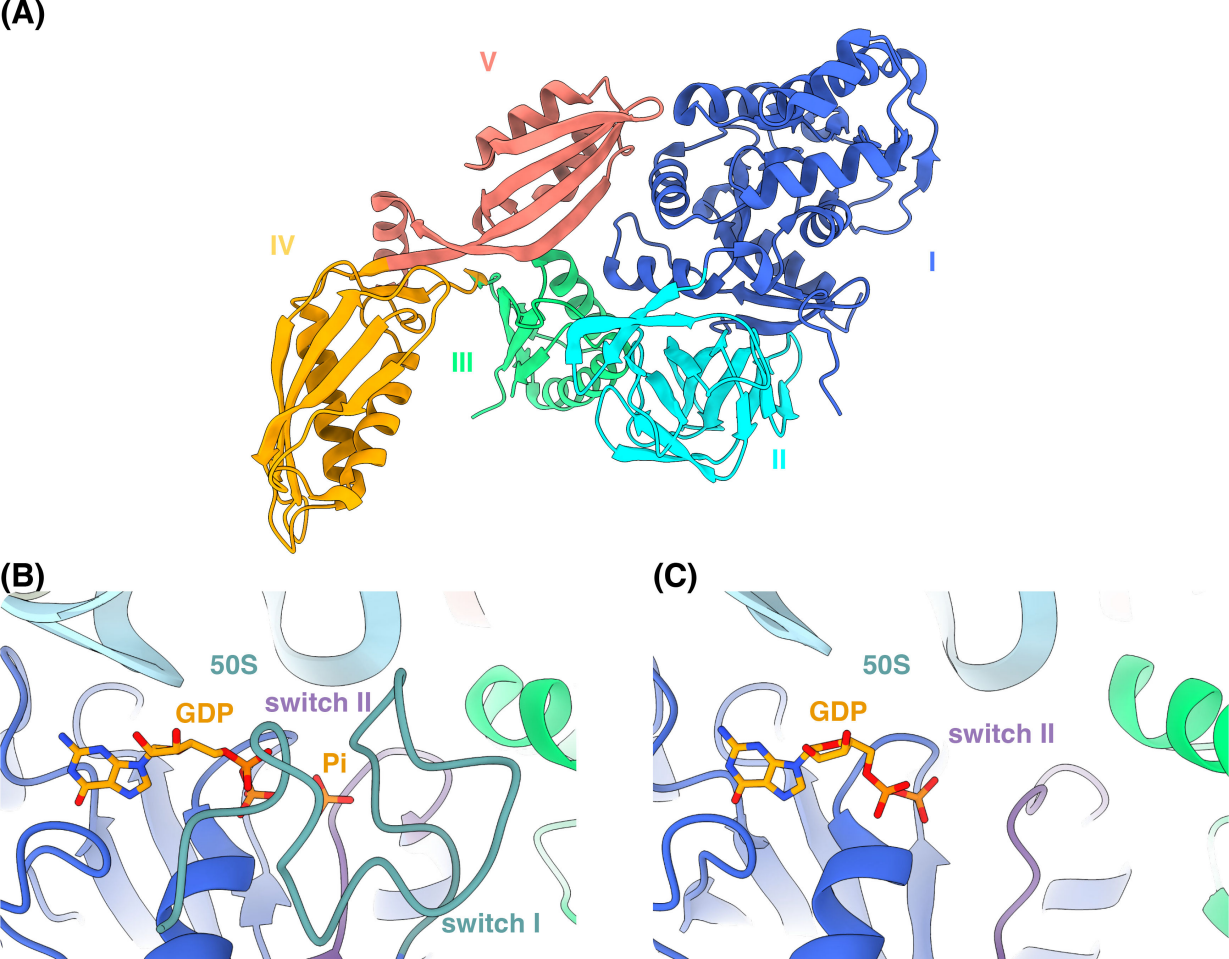
Structure of EF-G and its switch regions. (**A**) Crystal structure of *S. aureus* EF-G (PDBID: 2XEX) [15]. (**B**) Binding site of GDP and Pi (inorganic phosphate) in the cryo-EM structure of the *E. coli* ribosome with EF-G after GTP hydrolysis but before translocation (PDBID: 7SSL) [16]. Both switch I and switch II remain ordered and interact with Pi. (**C**) Binding site of GDP in the cryo-EM structure of the *E. coli* ribosome with EF-G after Pi release (PDBID: 7SSD) [16]. Upon Pi release, switch I becomes disordered and switch II moves away. EF-G is colored by domain and switch regions (I, blue; II, cyan; III, green; IV, orange; V, salmon red; switch I, blue-green, and switch II, purple), 50S subunit in blue, and GDP and Pi in orange.

**Figure 3:**
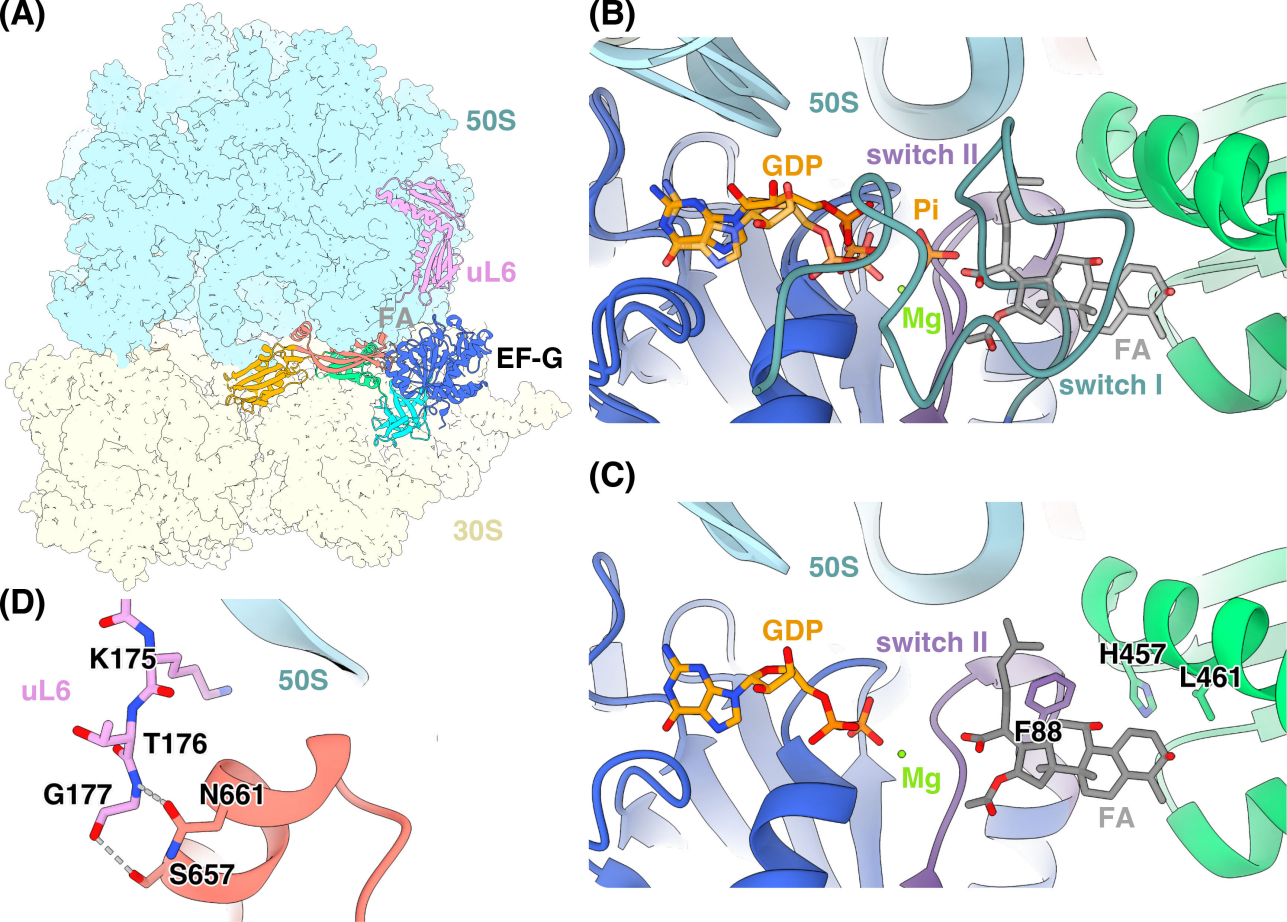
Structure of FA-locked EF-G on the ribosome. (**A**) Cryo-EM structure of *S. aureus* FA-locked 70S ribosome (PDBID: 8P2H) [18]. (**B**) Overlay of an *E. coli* 70S-EF-G complex with GDP•Pi (PDBID: 7SSL) [16] and *S. aureus* FA-locked 70S ribosome (PDBID: 8P2H [18], semi-transparent). FA can only bind when switch I is disordered. (**C**) FA binding site in the structure shown in panel A. Sites of *fusA* mutations mentioned in the text are shown as sticks. (**D**) Interaction between the C-terminus of uL6 and domain V of EF-G in the *S. aureus* FA-locked 70S ribosome (PDBID: 8P2H) [18]. The ribosome is colored by subunit (30S, yellow and 50S, blue), tRNA in beige, EF-G is colored by domain and switch regions (I, blue; II, cyan; III, green; IV, orange; V, salmon red; switch I, blue-green, and switch II, purple), GDP and Pi in orange, uL6 in light purple, FA in gray and magnesium in bright green.

EF-G is a five-domain protein (Figure 2A) [15] with two conserved switch regions (I and II, Figure 2B–C) that change their conformation after hydrolysis of GTP to GDP and release of inorganic phosphate. The conformational change of the switch regions is a prerequisite for the release of EF-G from the ribosome [16,17]. Structures of FA-inhibited ribosomes (Figure 3) show that FA binds next to the GDP binding site of EF-G (Figure 3B–C), in a pocket formed by domains I-III of EF-G and the 23S rRNA [18,19]. FA can only bind to EF-G after GTP hydrolysis on the ribosome, when switch I becomes disordered (Figure 3B), while it prevents the conformational change of switch II that would allow EF-G release [19]. During ribosome recycling, FA inhibition similarly prevents the conformational changes required to complete ribosome splitting and EF-G dissociation [14,20], but structural details are lacking.

### FA resistance through mutations in EF-G (FusA-type)

Mutations in *fusA*, the gene encoding EF-G, can provide resistance to FA, with MICs ranging from 2 to 256 µg/ml [21–23]. They are thought to occur spontaneously, leading to the appearance of resistant cells at low rates [24], and can give rise to resistance during the course of an antibiotic treatment.

Since FA binding and inhibition of EF-G only occurs after GTP hydrolysis on the ribosome, many mutations that directly or indirectly affect this ribosome-bound EF-G conformation will also perturb the FA-binding pocket. Thus, *fusA* resistance mutations have been classified according to their predicted effect into four groups: affecting EF-G-FA interactions, ribosome-EF-G interactions, EF-G conformation, and EF-G stability [15]. The highest-level single-substitution resistance mutations occur in amino acid positions shaping the FA binding pocket and making direct contacts with FA (F88L, H457Y, and L461K) (Figure 3C) [21,22,25]. Based on the analysis of FA-inhibited ribosome structures, these mutations are all predicted to directly perturb EF-G-FA interactions. Single resistance mutations further away from the FA pocket typically show lower level resistance. Many resistance mutations cluster in domain III of EF-G and are predicted to perturb the stability of this domain, its interactions with FA, or the conformational dynamics and locking of EF-G [15,18].

FusA-type resistance usually has a fitness cost as the function of EF-G is often disturbed by these mutations [21]. This is particularly the case for high-level resistance mutations in functionally important regions. However, *S. aureus* can acquire additional fitness-compensatory *fusA* mutations [21,26], which sometimes are far away in the sequence and structure. For this reason, it is often not mechanistically understood how they restore EF-G functionality, but one example has been elucidated in detail. F88 in switch II has a critical role in FA inhibition, as well as in transmission of conformational change in EF-G after GTP hydrolysis. It forms a hydrophobic contact with the dimethyl alkene end of FA and shapes the binding pocket through interaction with domain III (Figure 3C). The F88L mutation thus gives strong resistance but has a large fitness effect. The compensating M16I mutation in the core of domain I restores EF-G activity in translocation through facilitating inter-domain conformational change [27]. Another example is resistance mutation H457Y at the FA-facing side of domain III, which is predicted to de-stabilize the FA pocket and domain III. The fitness cost of this mutation has been shown to partially recover with the substitution S416F at the opposite edge of domain III [26], but the detailed mechanism of fitness loss and compensation remains unclear. It is unknown whether the mutation L461K also has a fitness effect, but structure analysis suggests that it may disturb FA binding without destabilizing domain III.

### FA resistance through target protection (FusB-type)

*S. aureus* can harbor additional genetic elements that provide resistance to FA. The first FA resistance plasmid, pUB101, was identified in 1976 [28]. This plasmid was later shown to encode a protein, FusB, that is responsible for the resistance through binding to EF-G [29]. FusB homologs encoded in the genome of some *S. aureus* isolates (FusC), *S. saprophyticus* (FusD) [30], and *S. cohnii* (FusF) [31] have also been identified. FusB and its homologs provide low-level resistance to FA (hereafter FusB-type resistance), with MICs in the range of 1–32 µg/ml FA [30,31]. FusB can provide rescue from FA inhibition *in vitro* during both translocation [29] and ribosome recycling [32]. The crystal structures of FusB [32] and FusC [33] are highly similar, consisting of two domains: an N-terminal α-helical bundle domain and a C-terminal treble-clef zinc finger domain. FusB-type resistance has been classified as *target protection* [34], since FusB binds with high affinity (dissociation constant, K_d_, of ~63 nM) to EF-G [33], the target of FA, to provide resistance through physical interaction without causing any permanent change to EF-G or FA. Nuclear magnetic resonance studies of a FusB complex with domains III-V of EF-G revealed that FusB causes increased dynamics of domain III of EF-G, and it was proposed that allosteric effects on the dynamics of EF-G would cause rescue from the steric block of FA [35].

**Figure 4:**
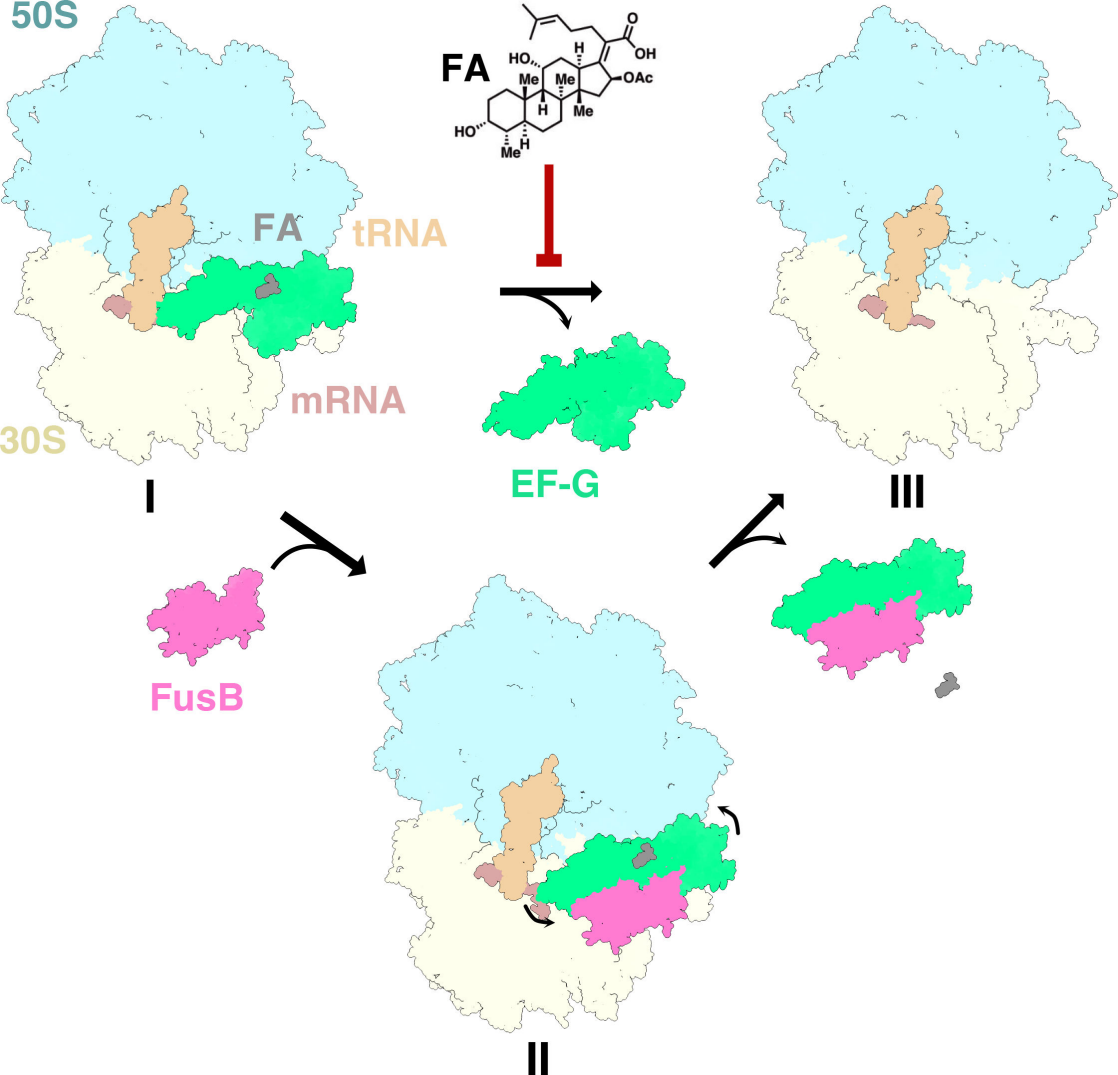
Mechanism of FusB-type resistance. (**I**) FA-inhibited ribosome. (**II**) FusB binds to EF-G on the FA-locked ribosome and causes major conformational changes that induce EF-G release followed by FA dissociation from EF-G. (**III**) Rescued ribosome ready to continue translation. The ribosome is colored by subunit (30S, yellow and 50S, blue), mRNA in brown, tRNAs in beige, EF-G in green, FA in gray, and FusB in pink.

Using cryo-EM, the transient rescue complex of FusB together with EF-G and FA on the 70S ribosome was recently captured [36], elucidating the mechanism of rescue (Figure 4). In this structure, FusB forms interactions with EF-G and the ribosome, causing major conformational changes of the FA-locked EF-G. FusB pushes EF-G domains I-II apart from domains IV-V and pulls domain IV away from the tRNA and mRNA. Domain III shows no signs of increased dynamics, moves along with domains I-II, and remains ordered. In this rescue state, FA remains bound, showing that EF-G release from the ribosome happens before dissociation of FA. The release of EF-G with FA from the ribosome differentiates FusB-type FA resistance from other target protection mechanisms [36], where the resistance proteins (e.g. TetO, TetM, or ABC-F proteins) directly promote dissociation of their target antibiotics to provide resistance [34]. A detailed understanding of how the dynamics of domain III in the FusB-EF-G complex [35] may contribute to FA resistance is still lacking. A mutated version of EF-G designed to restrict the dynamics of domain III could not be rescued by FusB [35], but it remains unclear whether this mutant remains functional in translocation and allows FusB binding.

Surprisingly, cryo-EM also showed binding of FusB directly to the 70S ribosome, in two distinct parts of the ribosomal A-site: mainly interacting with the 30S or 50S subunit. These binding sites overlap with the canonical binding site of EF-G, but it remains unclear whether the FusB-70S interaction (K_D_ of ~320 nM) [36] is linked to FA resistance or has some other function.

*In vitro*, FusB has been shown to decrease the residence time of EF-G on the ribosome, in absence as well as in presence of FA, and to cause inhibition of translation at high FusB concentrations [33]. This suggests that FusB-type resistance can also have a fitness cost and explains why expression of FusB from pUB101 is regulated by translational attenuation. Here, the alternative mRNA structure that allows FusB expression is only formed in the presence of FA, when FA-inhibited ribosomes stall during translation of a leader sequence [29]. Under inducing conditions in *S. aureus*, this can result in FusB production at a molar ratio of 1:2–3 to EF-G and low µM concentration of FusB [36]. It remains unknown how expression of FusB homologs is regulated.

### FA resistance through mutations in ribosomal protein uL6 (FusE-type)

Through laboratory selection of FA-resistant variants of *S. aureus,* a new class of resistance to FA was identified and designated FusE [22]. FusE variants carry mutations in the *rplF* gene that encodes ribosomal protein uL6. These are usually deletions leading to C-terminal truncations and provide low-level FA resistance with MICs of 4 µg/ml. FusE-class mutations come at a fitness cost to the cell, with >2-fold increased doubling times [22]. Structures of the FA-inhibited *S. aureus* 70S ribosome showed that the C-terminus of uL6 is in direct contact with EF-G (Figure 3D). Thus, these truncations may provide resistance by de-stabilizing the FA-locked state [18], similar to *fusA* mutations that affect EF-G–ribosome interactions.

### FA resistance in Gram-negative bacteria

The outer membrane of Gram-negative bacteria has limited permeability to FA [37] and harbors resistance-mediating efflux pumps. In *E. coli,* one such example is AcrAB-TolC, a multidrug efflux transport system that has been shown to bind and transport FA [38,39]. In fact, mutations [39] or knockout [40] of AcrAB-TolC make *E. coli* susceptible to FA. For these reasons, new FA formulations have potential for increased activity against Gram-negative bacteria if they address problems with permeability and AcrB binding. Thus, a FA-phospholipid complex has been shown to increase the permeability and efficacy of FA to *E. coli* and *Pseudomonas aeruginosa* [41]. Another interesting strategy is to combine FA with other antibiotics or substances that affect the outer membrane of Gram-negatives, such as polymyxin B [42], or the cell-penetrating peptide IMT-P8 [43].

### Additional mechanisms of FA resistance

Besides the clinically relevant mechanisms of FA resistance, additional resistance mechanisms of different types have been described in other organisms. They also risk to potentially emerge in staphylococci in the future.

Some streptomycetes secrete an extracellular esterase (FusH) that provides resistance to FA by hydrolyzing its ester bond [44]. There are also streptomycetes that produce ATP-binding cassette multidrug efflux pumps that, among other antibiotics, can provide resistance to FA [45].

In enterobacteria, a chloramphenicol acetyltransferase was suggested to potentially provide resistance through sequestering of FA [46,47].

### Prevalence of the mechanisms of resistance

**Figure 5:**
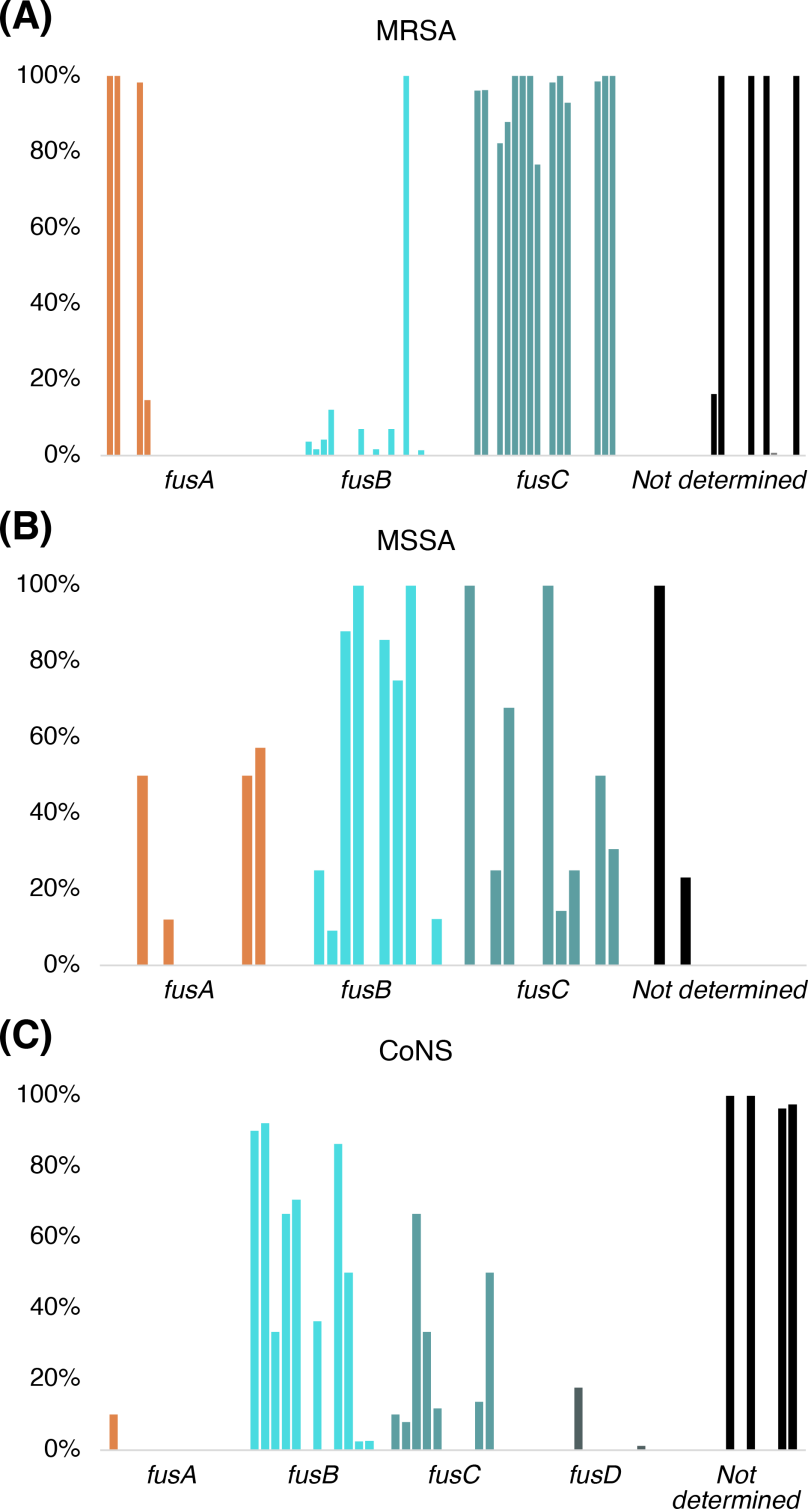
Percentage of isolates with each genetic resistance determinant in articles published in the last 5 years (2019–2025, data in Supplementary Table S1 **).** Each study is represented by one bar in each category of resistance determinant (*fusA*, *fusB*, *fusC*, *fusD,* or not determined). (**A**) MRSA isolates. (**B**) Methicillin-susceptible *S. aureus* (MSSA) or undetermined methicillin susceptibility. (**C**) Coagulase-negative staphylococci (CoNS).

Even though resistance to FA develops quickly *in vitro* due to *fusA* mutations [48], at a global scale, FA resistance was reported at an average prevalence of only ~5% in 2021 [49]. To further examine the relevance of each resistance mechanism, we have compiled studies published in the last 5 years where resistance mechanisms of isolates are specified [50–98] (Supplementary Table S1). Importantly, not all studies included analysis of all determinants. The compiled data show that FusB-type (FusB/C/D/F) resistance was the most prevalent (Figure 5). Interestingly, some clinical isolates have multiple *fusA* mutations, possibly indicating fitness-compensatory mutations. Others combine multiple resistance mechanisms, but this seems rare.

Of the *fusA* mutations, L461K was the most common, providing high-level resistance with MIC≥128 µg/ml, at least 2-fold above the other common mutation H457Q/Y and L461S. As discussed above, these mutations are expected to affect FA binding. We only found one case of *fusE* mutation reported in this time span [82]. However, the specific mutation (K101E) is unlikely to affect EF-G binding and was observed in combination with the high-level *fusA* resistance mutation L461K.

The prevalence of different types of resistance can relate to their respective fitness costs [22], as well as to how they spread and emerge. FusA-type and FusE-type resistances emerge from spontaneous mutations and come at a fitness cost. In contrast, the more prevalent FusB-type resistance can be horizontally transferred, e.g. by plasmids (FusB) [28], or staphylococcal cassette chromosome elements (FusC) [99].

### Future perspectives

FA is occasionally used in combination with other antibiotics, particularly rifampicin, to reduce resistance development [100]. However, it has been shown that rifampicin can induce metabolic transformation of FA, lowering its concentration, which may affect treatment [101,102]. Other studies show potential for different combinations, such as with linezolid, daptomycin, or vancomycin against staphylococcal biofilms [103]. Furthermore, sub-inhibitory concentrations of FA have been shown to reduce the virulence of *S. aureus* by reducing biofilm formation and toxin expression [104]. The potential of combination therapy is further supported by the observation that resistance mutations to other antibiotics can increase the sensitivity to FA [48].

FA has also been shown to be effective against pathogens outside its current clinical use. For example, it shows MIC values of ≤4 µg/ml in *Mycoplasma genitalia* [105], 0.5 µg/ml in *Mycobacterium tuberculosis* [106], and ≤8 µg/ml in some *Enterococcus spp* [107]. In addition, FA has been suggested to inhibit other targets in the cell, such as FtsZ, a cell division protein in *M. tuberculosis* [108] and efflux pumps in *Candida albicans* [109].

*S. aureus*, particularly MRSA, is a global threat; hence, new treatment options are essentially required. The global FA resistance rates are estimated to be low (~5%), particularly for MRSA (~2.6%) [49], supporting the value of FA as a treatment alternative. FA is currently available and used in many countries (including the EU, Australia, and the UK), but it is still not available in, for example, the United States, where the prevalence of FA resistance appears to be low (~3.7%) [49]. Our literature analysis shows that current FA resistance is primarily FusB-type, which can be horizontally transferred. Thus, surveillance and control will be important to reduce the spread of resistance and allow FA to remain effective.

**Perspectives**

- Antibiotic resistance is a global threat that needs to be addressed in many different ways. Fusidic acid (FA) is an antibiotic that in its clinical use targets protein synthesis in staphylococci. This mini-review aims to summarize the current knowledge regarding resistance mechanisms to FA and their prevalence.
- There are three types of FA resistance in staphylococci, involving target protection (FusB-type) or target mutagenesis (*fusA* and FusE). FusB-type resistance is the most common in clinical isolates. These main resistance mechanisms are mechanistically reasonably well understood.
- FA remains effective against staphylococcal infections, but surveillance and control will be important to reduce the spread of resistance. Its use could in the future potentially be expanded to other pathogens or improved through combination therapy.

## Supplementary Material

Online supplementary table 1

## Abbreviations

CoNS: Coagulase-negative staphylococci
EF-G: elongation factor G
FA: Fusidic acid
MIC: minimum inhibitory concentration
MRSA: methicillin-resistant *S. aureus*
MSSA: Methicillin-susceptible *S. aureus*
