# Supplementary material for: Mechanisms of fusidic acid resistance
Source: Biochem Soc Trans. 2025 Aug 18;53(4):1–12. doi: 10.1042/BST20253064 (PMC12493156; doi:10.1042/BST20253064)
Supplement: Online supplementary table 1 [file bst-53-04-BST20253064-s001.docx]

**Supplementary Table 1.** Genetic determinants of resistance from publications in the last five years (2019-2025). Abbreviations: MRSA (methicillin-resistant *Staphylococcus aureus*), MSSA (methicillin-susceptible *S. aureus*), MRSE (methicillin-resistant *S. epidermidis*), MSSE (methicillin-susceptible *S. epidermidis*), MRSP (methicillin-resistant *S. pseudintermedius*), MSSP (methicillin-susceptible *S. pseudintermedius*), CoNS (coagulase-negative staphylococci), ND (not determined), NS (not specified), MIC (minimum inhibitory concentration).

| Cell | Source | Country | Number of isolates | Genetic determinant (mutation) | MIC | Reference |
| --- | --- | --- | --- | --- | --- | --- |
| MRSA | Clinical isolate | USA | 4 | *fusA* (V90A, L461S) | NS | [1] |
| MRSA | Clinical isolate | China | 3 | *fusA* (H457Q) | NS |  |
| MRSA | Clinical isolate | Netherlands | 28 | *fusC* | NS | [2] |
| MRSA | Clinical isolate | Egypt | 25 | *fusC* | NS | [3] |
|  |  |  | 1 | ND | NS |  |
|  |  |  | 4 | Susceptible | NS |  |
| MRSA | Clinical isolate | New Caledonia | 21 | Susceptible | NS | [4] |
|  |  |  | 1 | *fusB* | 4 |  |
|  |  |  | 21 | *fusC* | 8 |  |
|  |  |  | 5 | *fusC* | 16 |  |
| MRSA | Clinical isolate | China | 383 | Susceptible | NS | [5] |
|  |  |  | 54 | *fusA* (L461K) | >128 |  |
|  |  |  | 3 | *fusA* (H457Q) | 2-16 |  |
|  |  |  | 1 | *fusA* (H457Y) | 32-64 |  |
|  |  |  | 1 | *fusA* (L461S) | 2-16 |  |
|  |  |  | 1 | *fusB* | 2-16 |  |
| MRSA | Clinical isolate | Kuwait | 4 | *fusB* | 4 | [6] |
|  |  |  | 76 | *fusC* | 4 |  |
|  |  |  | 1 |  | 12 |  |
|  |  |  | 1 |  | 16 |  |
|  |  |  | 1 | *fusA* (L461K), *fusC* | 256 |  |
|  |  |  | 13 | *fusA* (L461K) | 256 |  |
| MRSA | Clinical isolate | United Arab Emirates | 25 | *fusB* | NS | [7] |
|  |  |  | 181 | *fusC* | NS |  |
| MRSA | Clinical isolate | Kuwait | 42 | *fusC* | NS | [8] |
| MSSA | Clinical isolate | United Arab Emirates | 1 | *fusC* | NS | [9] |
| MRSA | Clinical isolate |  | 7 | *fusC* | NS |  |
| MRSA | Clinical isolate | Saudi Arabia | 5 | *fusC* | NS | [10] |
| MRSA | Clinical isolate | United Arab Emirates | 1 | *fusC* | NS |  |
| MRSA | Clinical isolate | Kuwait | 16 | *fusC* | NS |  |
| MRSA | Clinical isolate | Germany | 1 | *fusC* | NS |  |
| MRSA | Clinical isolate | Kuwait | 17 | Susceptible | NS | [11] |
|  |  |  | 33 | *fusC* | NS |  |
|  |  |  | 3 | *fusB* | NS |  |
|  |  |  | 7 | not *fusB/C* | NS |  |
| MRSA | Wild ungulates | Spain | 1 | Susceptible | NS | [12] |
|  |  |  | 6 | not *fusB/C* | NS |  |
| MRSA | Clinical isolate | Kuwait | 64 | Susceptible | NS | [13] |
|  |  |  | 116 | *fusC* | NS |  |
|  |  |  | 2 | *fusB* | NS |  |
| MRSA | Clinical isolate | Iran | 19 | Susceptible | NS | [14] |
|  |  |  | 1 | *fusC* | 32 |  |
| MRSA | Clinical isolate | Kuwait | 2,325 | *fusC* | NS | [15] |
|  |  |  | 177 | *fusB* | NS |  |
|  |  |  | 2224 | Susceptible | NS |  |
| MRSA | Clinical isolate | Portugal | 14 | Susceptible | NS | [16] |
|  |  |  | 2 | not *fusB/C* | NS |  |
| MRSA | Clinical isolate | Iran | 86 | Susceptible | NS | [17] |
|  |  |  | 3 | *fusB* | NS |  |
| MRSA | Clinical isolate | Spain | 134 | Susceptible | NS | [18] |
|  |  |  | 3 | not *fusB* | NS |  |
| MRSA | Clinical isolate | Kuwait | 1 | *fusB* | NS | [19] |
|  |  |  | 1 | *fusB* and *fusC* | NS |  |
|  |  |  | 141 | *fusC* | NS |  |
|  |  |  | 1 | not *fusB/C* | NS |  |
|  |  |  | 57 | Susceptible | NS |  |
| *S. aureus* | Bat guano | Algeria | 7 | Susceptible | NS | [20] |
| MRSA |  |  | 4 | *fusC* | NS |  |
| MRSA | Clinical isolate | United Arab Emirates | 14 | *fusC* | NS | [21] |
| MSSA |  |  | 8 | *fusC* | NS |  |
| MRSA | Community | Afganistan | 19 | Susceptible | NS | [22] |
| MSSA |  |  | 29 | Susceptible | NS |  |
|  |  |  | 2 | not *fusB* | NS |  |
| MSSA | Clinical isolate | Sicily | 1 | *fusB* | 8 | [23] |
|  |  |  | 1 | *fusA* (ND) | 8 |  |
|  |  |  | 1 | *fusA* (ND) | 16 |  |
|  |  |  | 1 | *fusC* | 32 |  |
| MSSA | Clinical isolate | Kuwait | 82 | *fusC* | NS | [24] |
|  |  |  | 11 | *fusB* | NS |  |
|  |  |  | 28 | ND | NS |  |
|  |  |  | 325 | Susceptible | NS |  |
| MSSA | Clinical isolate | Belgium | 3 | *fusA* (L461K) | NS | [25] |
|  |  |  | 29 | *fusB* | NS |  |
|  |  |  | 1 | *fusA* (H457Y) | NS |  |
| MSSA | Clinical isolate | Greece | 19 | Susceptible | NS | [26] |
|  |  |  | 241 | *fusB* | NS |  |
| MSSA | Clinical isolate | Iran | 72 | Susceptible | NS | [27] |
|  |  |  | 2 | *fusC* | 8 |  |
|  |  |  | 1 | *fusC* | 32 |  |
| *S. aureus* | Clinical isolate | Iran | 119 | Susceptible | NS | [28] |
|  |  |  | 6 | *fusB* | NS |  |
|  |  |  | 1 | *fusC* | 32 |  |
| *S. aureus* | Clinical isolate | Iran | 82 | Susceptible | NS | [29] |
|  |  |  | 3 | *fusB* | NS |  |
|  |  |  | 1 | *fusC* | NS |  |
| *S. aureus* | Clinical isolate | Spain and Italy | 16 | Susceptible | NS | [30] |
|  |  |  | 1 | *fusB* | NS |  |
| *S. aureus* | Clinical isolate | Portugal | 32 | Susceptible | NS | [31] |
|  |  |  | 1 | *fusC* | NS |  |
|  |  |  | 1 | *fusA* (A71V, H457Q, G476C) | NS |  |
| *S. aureus* | Bovine | Germany | 40 | Susceptible | NS | [32] |
| *S. aureus* | Cattle, pig and chicken carcasses | South Korea | 3,793 | Susceptible | NS | [33] |
|  |  |  | 2 | *fusA* (L461S) | 2 |  |
|  |  |  | 1 | *fusA* (L461K) | 256 |  |
|  |  |  | 98 | *fusA* (L461K) | 512 |  |
|  |  |  | 17 | *fusB* | 4 |  |
|  |  |  | 5 | *fusB* | 8 |  |
|  |  |  | 10 | *fusC* | 4 |  |
|  |  |  | 35 | *fusC* | 8 |  |
|  |  |  | 10 | *fusC* | 16 |  |
|  |  |  | 2 | *fusA* (L461K), *FusE* (K101E) | 512 |  |
| MRSA | Pork | South Korea | 2 | Susceptible | NS | [34] |
| MSSA |  |  | 2 | Susceptible | NS |  |
| *S. epidermidis* |  |  | 7 | *fusB* | 8-32 |  |
|  |  |  | 1 | *fusB* and *fusC* | 8 |  |
|  |  |  | 1 | *fusB* and *fusA* (V599I) | 24 |  |
|  |  |  | 1 | *fusB* | 0.25 |  |
| *Mammaliicoccus sciuri* |  |  | 32 | *fusB* homolog | 8-24 |  |
| MRSE and MSSE | Clinical isolate | China | 656 | Susceptible | NS | [35] |
|  |  |  | 47 | *fusB* | 4-32 |  |
|  |  |  | 4 | *fusC* | 4-32 |  |
| MRSE | Goat and farm | Saudi Arabia | 1 | *fusB* | NS | [36] |
|  |  |  | 2 | *fusC* | NS |  |
| MRSE | Clinical isolate | Sudan | 2 | *fusB* | NS | [37] |
|  | Clinical isolate |  | 1 | *fusC* | NS |  |
| MSSP | Dog isolate | Korea | 1 | *fusA* (A376V, P404L) | 8 | [38] |
| MSSP |  |  | 11 | Susceptible | NS |  |
| MRSP |  |  | 1 | *fusA* (V90I) | 6 |  |
|  |  |  | 1 | *fusA* (V90I) | 4 |  |
|  |  |  | 1 | ND | 8 |  |
|  |  |  | 1 | *fusA* (I461T) | 4 |  |
|  |  |  | 1 | ND | 2 |  |
|  |  |  | 1 | ND | 6 |  |
|  |  |  | 4 | *fusC* | 8 |  |
|  |  |  | 1 | *fusC* | 12 |  |
|  |  |  | 1 | *fusC* | 16 |  |
|  |  |  | 1 | *fusA* (I61Y, T62S), *fusC* | 12 |  |
|  |  |  | 27 | Susceptible | NS |  |
| MRSP | Dog isolate | UK | 3 | *fusC* | NS | [39] |
| CoNS |  | UK | 12 | *fusB* | NS |  |
| CoNS |  | UK | 2 | *fusD* | NS |  |
| CoNS |  | Romania | 2 | *fusC* | NS |  |
| CoNS |  | Romania | 1 | *fusD* | NS |  |
| CoNS | Food | Poland | 24 | *fusB/C/D* | NS | [40] |
|  |  |  | 61 | Susceptible | NS |  |
| CoNS | Wild Northeast Atlantic hakes | Northeast Atlantic Ocean | 16 | Susceptible | NS | [41] |
|  |  |  | 4 | *fusB* | NS |  |
|  |  |  | 7 | not *fusB* | NS |  |
| CoNS | Poultry | Portugal | 100 | Susceptible | NS | [42] |
|  |  |  | 120 | not *fusB/C/D* | NS |  |
| CoNS | Community | Germany | 105 | Susceptible | NS | [43] |
|  |  |  | 19 | *fusB* | NS |  |
|  |  |  | 3 | *fusC* | NS |  |
| CoNS | Cattle | Egypt | 2 | *fusB* | NS | [44] |
|  |  |  | 2 | *fusC* | NS |  |
|  |  |  | 71 | Susceptible | NS |  |
| CoNS | Wild birds | Spain | 24 | Susceptible | NS | [45] |
|  |  |  | 159 | not *fusB/C/D* | NS |  |
|  |  |  | 4 | *fusB* | NS |  |
|  |  |  | 2 | *fusD* | NS |  |
| CoNS | Wild rabbit | Portugal | 39 | Susceptible | NS | [46] |
|  |  |  | 1 | *fusB* | NS |  |
|  |  |  | 38 | not *fusB/C* | NS |  |
| *S. coagulans* | Dog | Portugal | 3 | not *fusB/C* | NS | [47] |
|  |  |  | 25 | Susceptible | NS |  |
| Clostridioides difficile | Clinical isolate | Mexico | 83 | Susceptible | NS | [48] |
|  |  |  | 7 | *fusA* (E117K) | NS |  |
|  |  |  | 4 | ND | NS |  |
| *Macrococcus spp.* | Calves, pig, beef and pork | Switzerland | 9 | *fusC* | NS | [49] |

**SUPPLEMENTARY REFERENCES**

1 Bonn, C.M., Rafiqullah, I.M., Crawford, J.A., Qian, Y.M., Guthrie, J.L., Matuszewska, M., et al. (2023) Repeated Emergence of Variant TetR Family Regulator, FarR, and Increased Resistance to Antimicrobial Unsaturated Fatty Acid among Clonal Complex 5 Methicillin-Resistant *Staphylococcus aureus*. *Antimicrob Agents Chemother* https://doi.org/10.1128/aac.00749-22

2 Vendrik, K.E.W., Kuijper, E.J., Dimmendaal, M., Silvis, W., Denie-Verhaegh, E., de Boer, A., et al. (2022) An unusual outbreak in the Netherlands: community-onset impetigo caused by a meticillin-resistant *Staphylococcus aureus* with additional resistance to fusidic acid, June 2018 to January 2020. *Eurosurveillance* **27** https://doi.org/10.2807/1560-7917.ES.2022.27.49.2200245

3 Monecke, S., Bedewy, A.K., Müller, E., Braun, S.D., Diezel, C., Elsheredy, A., et al. (2023) Characterisation of Methicillin-Resistant *Staphylococcus aureus* from Alexandria, Egypt. *Antibiotics* **12**, 78 https://doi.org/10.3390/antibiotics12010078

4 Bourles, A., Tristan, A., Vandenesch, F., Bes, M., Laurent, F., Ranc, A.-G., et al. (2022) A fusidic acid-resistant (PVL+) clone is associated with the increase in methicillin-resistant *Staphylococcus aureus* in New Caledonia. *J Glob Antimicrob Resist* **30**, 363–369 https://doi.org/10.1016/j.jgar.2022.07.005

5 Zhao, H., Wang, X., Wang, B., Xu, Y., Rao, L., Wan, B., et al. (2021) The Prevalence and Determinants of Fusidic Acid Resistance Among Methicillin-Resistant *Staphylococcus aureus* Clinical Isolates in China. *Front Med (Lausanne)* **8** https://doi.org/10.3389/fmed.2021.761894

6 Boloki, H.A., Al-Musaileem, W.F., AlFouzan, W., Verghese, T. and Udo, E.E. (2021) Fusidic Acid Resistance Determinants in Methicillin-Resistant *Staphylococcus aureus* Isolated in Kuwait Hospitals. *Medical Principles and Practice* **30**, 542–549 https://doi.org/10.1159/000518408

7 Senok, A., Nassar, R., Celiloglu, H., Nabi, A., Alfaresi, M., Weber, S., et al. (2020) Genotyping of methicillin resistant *Staphylococcus aureus* from the United Arab Emirates. *Sci Rep* **10**, 18551 https://doi.org/10.1038/s41598-020-75565-w

8 Udo, E.E., Boswihi, S.S., Mathew, B., Noronha, B., Verghese, T., Al-Jemaz, A., et al. (2020) Emergence of Methicillin-Resistant *Staphylococcus aureus* Belonging to Clonal Complex 15 (CC15-MRSA) in Kuwait Hospitals. *Infect Drug Resist* **13**, 617–626 https://doi.org/10.2147/IDR.S237319

9 Senok, A., Nassar, R., Kaklamanos, E.G., Belhoul, K., Abu Fanas, S., Nassar, M., et al. (2020) Molecular Characterization of *Staphylococcus aureus* Isolates Associated with Nasal Colonization and Environmental Contamination in Academic Dental Clinics. *Microbial Drug Resistance* **26**, 661–669 https://doi.org/10.1089/mdr.2019.0318

10 Senok, A., Slickers, P., Hotzel, H., Boswihi, S., Braun, S.D., Gawlik, D., et al. (2019) Characterisation of a novel SCCmec VI element harbouring *fusC* in an emerging *Staphylococcus aureus* strain from the Arabian Gulf region. *PLoS One* **14**, e0223985 https://doi.org/10.1371/journal.pone.0223985

11 Alfouzan, W.A., Boswihi, S.S. and Udo, E.E. (2023) Methicillin-Resistant *Staphylococcus aureus* (MRSA) in a Tertiary Care Hospital in Kuwait: A Molecular and Genetic Analysis. *Microorganisms* **12**, 17 https://doi.org/10.3390/microorganisms12010017

12 Rey Pérez, J., Zálama Rosa, L., García Sánchez, A., Hermoso de Mendoza Salcedo, J., Alonso Rodríguez, J.M., Cerrato Horrillo, R., et al. (2021) Multiple Antimicrobial Resistance in Methicillin-Resistant *Staphylococcus sciuri* Group Isolates from Wild Ungulates in Spain. *Antibiotics* **10**, 920 https://doi.org/10.3390/antibiotics10080920

13 Sarkhoo, E., Udo, E.E., Boswihi, S.S., Monecke, S., Mueller, E. and Ehricht, R. (2021) The Dissemination and Molecular Characterization of Clonal Complex 361 (CC361) Methicillin-Resistant *Staphylococcus aureus* (MRSA) in Kuwait Hospitals. *Front Microbiol* **12** https://doi.org/10.3389/fmicb.2021.658772

14 Tayebi, Z., Fazeli, M., Hashemi, A., Abdi, S., Dadashi, M., Nasiri, M.J., et al. (2021) Molecular characterization of invasive *Staphylococcus aureus* strains isolated from patients with diabetes in Iran: USA300 emerges as the major type. *Infection, Genetics and Evolution* **87**, 104679 https://doi.org/10.1016/j.meegid.2020.104679

15 Boswihi, S.S., Udo, E.E. and AlFouzan, W. (2020) Antibiotic resistance and typing of the methicillin-resistant *Staphylococcus aureus* clones in Kuwait hospitals, 2016–2017. *BMC Microbiol* **20**, 314 https://doi.org/10.1186/s12866-020-02009-w

16 Silva, V., Hermenegildo, S., Ferreira, C., Manaia, C.M., Capita, R., Alonso-Calleja, C., et al. (2020) Genetic Characterization of Methicillin-Resistant *Staphylococcus aureus* Isolates from Human Bloodstream Infections: Detection of MLSB Resistance. *Antibiotics* **9**, 375 https://doi.org/10.3390/antibiotics9070375

17 Goudarzi, M., Razeghi, M., Chirani, A.S., Fazeli, M., Tayebi, Z. and Pouriran, R. (2020) Characteristics of methicillin-resistant *Staphylococcus aureus* carrying the toxic shock syndrome toxin gene: high prevalence of clonal complex 22 strains and the emergence of new spa types t223 and t605 in Iran. *New Microbes New Infect* **36**, 100695 https://doi.org/10.1016/j.nmni.2020.100695

18 Ceballos, S., Aspiroz, C., Ruiz-Ripa, L., Zarazaga, M. and Torres, C. (2020) Antimicrobial resistance phenotypes and genotypes of methicillin-resistant *Staphylococcus aureus* CC398 isolates from Spanish hospitals. *Int J Antimicrob Agents* **55**, 105907 https://doi.org/10.1016/j.ijantimicag.2020.105907

19 Boswihi, S.S., Udo, E.E., Mathew, B., Noronha, B., Verghese, T. and Tappa, S.B. (2020) Livestock-Associated Methicillin-Resistant *Staphylococcus aureus* in Patients Admitted to Kuwait Hospitals in 2016–2017. *Front Microbiol* **10** https://doi.org/10.3389/fmicb.2019.02912

20 Mairi, A., Touati, A., Pantel, A., Yahiaoui Martinez, A., Ahmim, M., Sotto, A., et al. (2021) First Report of CC5-MRSA-IV-SCCfus “Maltese Clone” in Bat Guano. *Microorganisms* **9**, 2264 https://doi.org/10.3390/microorganisms9112264

21 Senok, A., Monecke, S., Nassar, R., Celiloglu, H., Thyagarajan, S., Müller, E., et al. (2021) Lateral Flow Immunoassay for the Detection of Panton-Valentine Leukocidin in *Staphylococcus aureus* From Skin and Soft Tissue Infections in the United Arab Emirates. *Front Cell Infect Microbiol* **11** https://doi.org/10.3389/fcimb.2021.754523

22 Naimi, H.M., Tristan, A., Bes, M., Vandenesch, F., Nazari, Q.A., Laurent, F., et al. (2023) Molecular characterization and antimicrobial resistance of nasal *Staphylococcus aureus* in the community of Kabul. *J Glob Antimicrob Resist* **34**, 18–22 https://doi.org/10.1016/j.jgar.2023.06.001

23 Bivona, D., Nicitra, E., Bonomo, C., Calvo, M., Migliorisi, G., Perez, M., et al. (2024) Molecular diversity in fusidic acid–resistant Methicillin Susceptible *Staphylococcus* *aureus*. *JAC Antimicrob Resist* **6** https://doi.org/10.1093/jacamr/dlae154

24 Boswihi, S.S., Alfouzan, W.A. and Udo, E.E. (2024) Genomic profiling of methicillin-sensitive *Staphylococcus aureus* (MSSA) isolates in Kuwait hospitals. *Front Microbiol* **15** https://doi.org/10.3389/fmicb.2024.1361217

25 Yin, N., Michel, C., Makki, N., Deplano, A., Milis, A., Prevost, B., et al. (2024) Emergence and spread of a mupirocin-resistant variant of the European epidemic fusidic acid-resistant impetigo clone of *Staphylococcus aureus*, Belgium, 2013 to 2023. *Eurosurveillance* **29** https://doi.org/10.2807/1560-7917.ES.2024.29.19.2300668

26 Giormezis, N., Doudoulakakis, A., Tsilipounidaki, K., Militsopoulou, M., Kalogeras, G., Stamouli, V., et al. (2021) Emergence of a mupirocin-resistant, methicillin-susceptible *Staphylococcus aureus* clone associated with skin and soft tissue infections in Greece. *BMC Microbiol* **21**, 203 https://doi.org/10.1186/s12866-021-02272-5

27 Goudarzi, H., Goudarzi, M., Sabzehali, F., Fazeli, M. and Salimi Chirani, A. (2020) Genetic analysis of methicillin‐susceptible *Staphylococcus aureus* clinical isolates: High prevalence of multidrug‐resistant ST239 with strong biofilm‐production ability. *J Clin Lab Anal* **34** https://doi.org/10.1002/jcla.23494

28 Goudarzi, M., Kobayashi, N., Dadashi, M., Pantůček, R., Nasiri, M.J., Fazeli, M., et al. (2020) Prevalence, Genetic Diversity, and Temporary Shifts of Inducible Clindamycin Resistance *Staphylococcus aureus* Clones in Tehran, Iran: A Molecular–Epidemiological Analysis From 2013 to 2018. *Front Microbiol* **11** https://doi.org/10.3389/fmicb.2020.00663

29 Goudarzi, M., Tayebi, Z., Fazeli, M., Miri, M. and Nasiri, M.J. (2020) Molecular Characterization, Drug Resistance and Virulence Analysis of Constitutive and Inducible Clindamycin Resistance *Staphylococcus aureus* Strains Recovered from Clinical Samples, Tehran – Iran. *Infect Drug Resist* **Volume 13**, 1155–1162 https://doi.org/10.2147/IDR.S251450

30 Cabrera, R., Fernández-Barat, L., Motos, A., López-Aladid, R., Vázquez, N., Panigada, M., et al. (2020) Molecular characterization of methicillin-resistant *Staphylococcus aureus* clinical strains from the endotracheal tubes of patients with nosocomial pneumonia. *Antimicrob Resist Infect Control* **9**, 43 https://doi.org/10.1186/s13756-020-0679-z

31 Ferreira, C., Costa, S.S., Serrano, M., Oliveira, K., Trigueiro, G., Pomba, C., et al. (2021) Clonal Lineages, Antimicrobial Resistance, and PVL Carriage of *Staphylococcus aureus* Associated to Skin and Soft-Tissue Infections from Ambulatory Patients in Portugal. *Antibiotics* **10**, 345 https://doi.org/10.3390/antibiotics10040345

32 Moawad, A.A., El-Adawy, H., Linde, J., Jost, I., Tanja, G., Katja, H., et al. (2023) Whole genome sequence-based analysis of *Staphylococcus aureus* isolated from bovine mastitis in Thuringia, Germany. *Front Microbiol* **14** https://doi.org/10.3389/fmicb.2023.1216850

33 Kim, S.-J., Ali, Md.S., Kang, H.-S., Moon, B.-Y., Hwang, Y.-J., Yoon, S.-S., et al. (2025) Characterization of fusidic acid-resistant *Staphylococcus aureus* isolated from food animals during 2010–2021 in South Korea. *Int J Food Microbiol* **430**, 111026 https://doi.org/10.1016/j.ijfoodmicro.2024.111026

34 Yang, Y.J., Lee, G.Y., Kim, S. Do, Park, J.H., Lee, S.I., Kim, G.-B., et al. (2022) Profiles of Non-aureus Staphylococci in Retail Pork and Slaughterhouse Carcasses: Prevalence, Antimicrobial Resistance, and Genetic Determinant of Fusidic Acid Resistance. *Food Sci Anim Resour* **42**, 225–239 https://doi.org/10.5851/kosfa.2021.e74

35 Chen, S., Rao, L. and Lin, C. (2022) The Dissemination of Fusidic Acid Resistance Among *Staphylococcus epidermidis* Clinical Isolates in Wenzhou, China. *Infect Drug Resist* **Volume 15**, 2537–2544 https://doi.org/10.2147/IDR.S365071

36 El-Deeb, W., Cave, R., Fayez, M., Alhumam, N., Quadri, S. and Mkrtchyan, H. V. (2022) Methicillin Resistant Staphylococci Isolated from Goats and Their Farm Environments in Saudi Arabia Genotypically Linked to Known Human Clinical Isolates: a Pilot Study. *Microbiol Spectr* **10** https://doi.org/10.1128/spectrum.00387-22

37 Altayb, H.N., Elbadawi, H.S., Baothman, O., Kazmi, I., Alzahrani, F.A., Nadeem, M.S., et al. (2022) Whole-Genome Sequence of Multidrug-Resistant Methicillin-Resistant *Staphylococcus epidermidis* Carrying Biofilm-Associated Genes and a Unique Composite of SCCmec. *Antibiotics* **11**, 861 https://doi.org/10.3390/antibiotics11070861

38 Lim, Y., Hyun, J. and Hwang, C. (2020) Identification of fusidic acid resistance in clinical isolates of *Staphylococcus pseudintermedius* from dogs in Korea. *Vet Dermatol* **31**, 267 https://doi.org/10.1111/vde.12844

39 Hritcu, O.M., Schmidt, V.M., Salem, S.E., Maciuca, I.E., Moraru, R.F., Lipovan, I., et al. (2020) Geographical Variations in Virulence Factors and Antimicrobial Resistance Amongst Staphylococci Isolated From Dogs From the United Kingdom and Romania. *Front Vet Sci* **7** https://doi.org/10.3389/fvets.2020.00414

40 Chajęcka-Wierzchowska, W., Gajewska, J., Zadernowska, A., Randazzo, C.L. and Caggia, C. (2023) A Comprehensive Study on Antibiotic Resistance among Coagulase-Negative Staphylococci (CoNS) Strains Isolated from Ready-to-Eat Food Served in Bars and Restaurants. *Foods* **12**, 514 https://doi.org/10.3390/foods12030514

41 Díaz-Formoso, L., Silva, V., Contente, D., Feito, J., Hernández, P.E., Borrero, J., et al. (2023) Antibiotic Resistance Genes, Virulence Factors, and Biofilm Formation in Coagulase-Negative *Staphylococcus spp.* Isolates from European Hakes (*Merluccius merluccius*, L.) Caught in the Northeast Atlantic Ocean. *Pathogens* **12**, 1447 https://doi.org/10.3390/pathogens12121447

42 Silva, V., Caniça, M., Ferreira, E., Vieira-Pinto, M., Saraiva, C., Pereira, J.E., et al. (2022) Multidrug-Resistant Methicillin-Resistant Coagulase-Negative Staphylococci in Healthy Poultry Slaughtered for Human Consumption. *Antibiotics* **11**, 365 https://doi.org/10.3390/antibiotics11030365

43 Marincola, G., Liong, O., Schoen, C., Abouelfetouh, A., Hamdy, A., Wencker, F.D.R., et al. (2021) Antimicrobial Resistance Profiles of Coagulase-Negative Staphylococci in Community-Based Healthy Individuals in Germany. *Front Public Health* **9** https://doi.org/10.3389/fpubh.2021.684456

44 El-Ashker, M., Gwida, M., Monecke, S., Ehricht, R., Elsayed, M., El-Gohary, F., et al. (2020) Microarray-based detection of resistance genes in coagulase-negative staphylococci isolated from cattle and buffalo with mastitis in Egypt. *Trop Anim Health Prod* **52**, 3855–3862 https://doi.org/10.1007/s11250-020-02424-1

45 Ruiz-Ripa, L., Gómez, P., Alonso, C.A., Camacho, M.C., Ramiro, Y., de la Puente, J., et al. (2020) Frequency and Characterization of Antimicrobial Resistance and Virulence Genes of Coagulase-Negative Staphylococci from Wild Birds in Spain. Detection of tst-Carrying *S. sciuri* Isolates. *Microorganisms* **8**, 1317 https://doi.org/10.3390/microorganisms8091317

46 Sousa, M., Silva, V., Silva, A., Silva, N., Ribeiro, J., Tejedor-Junco, M.T., et al. (2020) Staphylococci among Wild European Rabbits from the Azores: A Potential Zoonotic Issue? *J Food Prot* **83**, 1110–1114 https://doi.org/10.4315/0362-028X.JFP-19-423

47 Costa, S.S., Oliveira, V., Serrano, M., Pomba, C. and Couto, I. (2021) Phenotypic and Molecular Traits of *Staphylococcus coagulans* Associated with Canine Skin Infections in Portugal. *Antibiotics* **10**, 518 https://doi.org/10.3390/antibiotics10050518

48 Aguilar-Zamora, E., Weimer, B.C., Torres, R.C., Gómez-Delgado, A., Ortiz-Olvera, N., Aparicio-Ozores, G., et al. (2022) Molecular Epidemiology and Antimicrobial Resistance of *Clostridioides difficile* in Hospitalized Patients From Mexico. *Front Microbiol* **12** https://doi.org/10.3389/fmicb.2021.787451

49 Keller, J.E., Schwendener, S., Neuenschwander, J., Overesch, G. and Perreten, V. (2022) Prevalence and characterization of -methicillin-resistant *Macrococcus spp.* in food producing animals and meat in Switzerland in 2019. *Schweiz Arch Tierheilkd* **164**, 153–164 https://doi.org/10.17236/sat00343
